# The FACT-GP5 as a global tolerability measure: responsiveness and robustness to missing assessments

**DOI:** 10.1007/s11136-024-03740-x

**Published:** 2024-07-24

**Authors:** Cara Arizmendi, Yanyan Zhu, Maryam Khan, Jonathon Gable, Bryce B. Reeve, Bellinda King-Kallimanis, Jill Bell

**Affiliations:** 1 AstraZeneca, Oncology Digital Health R&D, Gaithersburg, MD USA; 2 Center for Health Measurement, Department of Population Health Sciences, Duke University School of Medicine, Durham, NC USA; 3 Lungevity Foundation, Chicago, IL USA

**Keywords:** Tolerability, Oncology, Patient-reported outcomes, FACT-GP5, Missing data, Trajectories

## Abstract

**Purpose:**

The Functional Assessment of Cancer Therapy item (FACT-GP5) has the potential to provide an understanding of global treatment tolerability from the patient perspective. Longitudinal evaluations of the FACT-GP5 and challenges posed by data missing-not-at-random (MNAR) have not been explored. Robustness of the FACT-GP5 to missing data assumptions and the responsiveness of the FACT-GP5 to key side-effects are evaluated.

**Methods:**

In a randomized, double-blind study (NCT00065325), postmenopausal women (*n* = 618) with hormone receptor-positive (HR+), advanced breast cancer received either fulvestrant or exemestane and completed FACT measures monthly for seven months. Cumulative link mixed models (CLMM) were fit to evaluate: (1) the trajectory of the FACT-GP5 and (2) the responsiveness of the FACT-GP5 to CTCAE grade, Eastern Cooperative Oncology Group (ECOG) Performance Status scale, and key side-effects from the FACT. Sensitivity analyses of the missing-at-random (MAR) assumption were conducted.

**Results:**

Odds of reporting worse side-effect bother increased over time. There were positive within-person relationships between level of side-effect bother (FACT-GP5) and severity of other FACT items, as well as ECOG performance status and Common Terminology Criteria for Adverse Events (CTCAE) grade. The number of missing FACT-GP5 assessments *impacted the trajectory* of the FACT-GP5 but *did not impact the relationships between* the FACT-GP5 and other items (except for nausea [FACT-GP2]).

**Conclusions:**

Results support the responsiveness of the FACT-GP5. Generally speaking, the responsiveness of the FACT-GP5 is robust to missing assessments. Missingness should be considered, however, when evaluating change over time of the FACT-GP5.

**Trial Registration:**

NCT00065325.

**Trial Registration Year:**

2003.

**Supplementary Information:**

The online version contains supplementary material available at 10.1007/s11136-024-03740-x.

## Plain language summary

Researchers have been exploring the use of a single question, FACT-GP5 (“*I am bothered by side effects of treatment*”), as a quick way to learn about drug tolerability from the patients’ perspective. This study explores if this single question can capture changes in tolerability during treatment, and if the assessment is missed by patients, whether that impacts the interpretation of tolerability. In our study, we found that the FACT-GP5 can be used to understand how tolerability changes during treatment. Missing assessments of the FACT-GP5 are important to account for when interpreting results. The FACT-GP5 may be a useful question for capturing the patient experience of drug tolerability.

As the treatment paradigm in oncology shifts away from the legacy of cytotoxic chemotherapy regimens with fixed treatment durations towards an era of increasingly selective targeted therapies with variable, often longer treatment durations, tolerability has become an increasingly critical component of how we evaluate and compare novel treatments [1]. Traditionally, adverse events have been directly reported by clinicians, however there is growing support for patients to self-report symptomatic adverse events on how well they may tolerate therapy [2]. The collection of patient experience data in trials provides a more comprehensive view of treatment benefit and risk profiles and allows patients and prescribers to make more informed treatment decisions [1, 3–5].

To capture overall tolerability from the patient perspective, global items, that capture a person’s perception of the impact of their side effects, have been recommended for use [6]. One such item is the Functional Assessment of Cancer Therapy - General Population item on severity of side-effect bother (FACT-GP5; “*I am bothered by side effects of treatment*”) [6–10].

## FACT-GP5

Researchers have presented the FACT-GP5 as a potential global tolerability measure because of its face validity [9]. Additionally, the FACT-GP5 is a single item, giving patients the opportunity to provide an overall rating of side-effect bother with little added burden. Having a global side-effect bother item simplifies the direct comparison of treatment arms. This may be especially useful where there are different side effect profiles in each study arm [7] or when patients consider specific side-effects to have greater salience to the overall tolerability of a drug [6]. Including the FACT-GP5 in the patient-reported outcome (PRO) strategy as a complimentary item to specific symptom data may help to overcome some of the complications associated with interpreting a summary of specific symptoms, when evaluating overall tolerability.

The FACT-GP5 has been found to be understandable and meaningful to people with cancer, particularly after treatment initiation [9]. Griffiths and colleagues [7] found that those reporting lower side-effect bother reported better global health, suggesting convergent validity. They also found higher side effect bother for patients with more advanced cancer stages and increased Eastern Cooperative Oncology Group (ECOG) Performance Scale status, suggesting known-groups validity. Similarly, Pearman and colleagues [8] found relationships between the FACT-GP5 and linked Common Terminology Criteria for Adverse Events (CTCAE) grades and inverse relationships with the FACT-GF3 (“I am able to enjoy life”) and the EuroQol 5D (EQ-5D) health utility score. Trask and colleagues [10] also found the FACT-GP5 to be sensitive to change and responsive across two timepoints, with improvement in physical well-being and physical-function being related to lower likelihood of greater side-effect bother. In addition, the FACT-GP5 has been used as a single-item exploratory outcome in renal cell carcinoma and prostate cancer clinical trials [11, 12]. Finally, several trials registered with clinicaltrials.gov include the FACT-GP5 as a secondary outcome [13–28].

## Current aims

The current study builds upon previous psychometric evaluations of the FACT-GP5 by examining:

1. The responsiveness of the FACT-GP5 via within-person relationships of the FACT-GP5 with CTCAE grade, ECOG performance status, and other FACT symptom items associated with the safety profile of study treatments.
2. The impact of missing assessments of the FACT-GP5 on the trajectory of the FACT-GP5 and the relationship of the FACT-GP5 with CTCAE grade, ECOG performance status, and other FACT items via pattern mixture model sensitivity analyses.

Understanding if the FACT-GP5 can be responsive will provide greater insight into its suitability as a global tolerability measure by exploring whether the FACT-GP5 can be used to detect change that is meaningful to patients. The current study extends the work of Griffiths et al. [7] Pearman et al. [8], and Trask et al. [10] by examining the responsiveness of the FACT-GP5 in relation to CTCAE grade, ECOG performance status, and FACT items relevant to the side-effect profiles of the study drugs over multiple timepoints.

Additionally, in cancer clinical trials, missing data are common, and it is likely that tolerability data are missing not at random (MNAR, i.e., the missing observations are related to unobserved data) [29], though this assumption for the FACT-GP5 item has not been empirically tested. Missing observations of the FACT-GP5 may be due to patients experiencing higher levels of side-effect bother. An empirical examination through pattern mixture models will provide insight as to how trajectories and responsiveness of the FACT-GP5 may differ under MNAR versus missing at random (MAR) assumptions.

## Methods

### Data

Data come from a randomized, double-blind, global, multicenter study (NCT00065325) [30] comparing the efficacy and tolerability of fulvestrant to exemestane after prior nonsteroidal aromatase inhibitor therapy in women who are postmenopausal with hormone receptor-positive (HR+) advanced or metastatic breast cancer. Inclusion required women to have a World Health Organization (WHO) performance status of 0 to 2, a life expectancy of at least three months, and at least one measurable or assessable lesion. Exclusion criteria included the presence of life-threating metastatic visceral disease, brain or leptomeningeal metastases, prior exposure to fulvestrant or exemestane, extensive radiation or cytotoxic therapy within the last four weeks, or a history of bleeding diathesis or need for long-term anticoagulation [30]. Patients (*n* = 693) across 138 worldwide centers were randomized 1:1 to either fulvestrant (*n* = 351) or exemestane (*n* = 342). All randomized patients who completed at least one FACT-GP5 assessment (*n* = 618) were included in the current analyses. Further details about the original study can be found in Chia et al. [30].

### Measures

Patients completed the Functional Assessment of Cancer Therapy – General (FACT-G), the FACT – Breast (FACT-B), and the FACT – Endocrine Symptom (FACT-ES) at baseline, monthly for six months, and every three months thereafter. We chose to look at the measurements from baseline and the first six months, as the available data of the total 618 patients for the FACT-GP5 had dropped to 36.89% (*n* = 228) at month six. Clinicians also completed the ECOG performance scale, a six-point scale indicating health status, on the same schedule of assessment. Finally, adverse events (AEs) were recorded using the CTCAE version 3.0 along with start and stop dates of the AE. Because we were interested in study drug tolerability, we restricted AEs in the analysis to those that were determined by clinicians to have a reasonable possibility of being related to study treatment.

### FACT items

The FACT-GP5 is a single item from the FACT that asks patients to rate the statement, “I am bothered by side effects of treatment,” on a five-point scale from 0 (“Not at all”) to 4 (“Very much”) “as it applies to the past seven days”. To explore the relationship of the FACT-GP5 to side-effect items relevant to the side-effect profile of the study drug, we selected other FACT-G and FACT-ES items for modeling based on the most commonly occurring treatment-related, clinician-reported AEs (> 5% incidence in both treatment groups) in the original study [30]. This led to the selection of (1) FACT-GP1 – “I have a lack of energy,” (2) FACT-GP2 – “I have nausea,” (3) FACT-GP4 – “I have pain,” and (4) FACT-ES1 – “I have hot flashes/flushes.” All items are rated on a five-point scale from 0 (“Not at all”) to 4 (“Very much”) as it applies to the past seven days.

#### Alignment of CTCAE Grade with FACT-GP5

Because CTCAE grade was not collected on the FACT schedule of assessments, it was necessary to align CTCAE grade to the same time scale as the FACT. Alignment of clinician-reported CTCAE grade with the FACT-GP5 was done using the onset of AE and AE stop dates recorded by the clinician. For AEs that were unresolved, the resolution date was coded as the last day of participation in the study. The value aligned with the patient-reported measures was the maximum CTCAE grade with a reasonable possibility of being caused by study treatment that occurred within the 7-day recall period of the patients’ FACT completion date. If clinicians did not report a treatment-related AE in the 7-day window, the CTCAE grade for that timepoint was coded as zero.

### Statistical analyses

#### Descriptive Summary

These analyses were a post-hoc exploratory evaluation. All analyses were completed using *R Statistical Software* (v 4.0.3) [31]. Descriptive statistics for patients’ age, race, ethnicity, ECOG performance status at baseline, and presence of measurable disease were calculated. Additionally, the mean and median were calculated for the FACT items of interest and ECOG performance status at each timepoint. Because the FACT-GP5 is treated as categorical in the statistical models, number of patients reporting each response option, with percentages, were reported for the FACT-GP5. Mean and median CTCAE grade were also calculated.

### Modeling approach

To address Aim 1, four separate cumulative linked mixed models (CLMMs), using the *ordinal* [32] package in R, were fit. For all models, the probability of a patient reporting each of the five levels of the FACT-GP5 was the dependent variable.

- Model 1 was a base model to gain insights into the trajectory of the FACT-GP5. Timepoint, coded from 1 (Baseline) to 7 (Month 6), was included as a predictor to model the trend but also to control for the trend in subsequent models. Controlling for time removes potential confounds of trends from the estimates of relationships between the FACT-GP5 and other variables in subsequent models (Models 2–4). [33].
- Model 2 built upon Model 1. CTCAE grade was added as a predictor, to examine the relationship between the FACT-GP5 and CTCAE grade, controlling for time.
- Model 3 built upon Model 1. ECOG performance status was added as a predictor to examine the relationship between the FACT-GP5 and ECOG performance status, controlling for time.
- Model 4 built upon Model 1. FACT item scores for the four selected side-effect items were added as predictors to look at the relationship between the FACT-GP5 and FACT item scores, controlling for time.

CTCAE grade, ECOG performance status, and the four FACT item predictors were person mean-centered to model the relationship between the FACT-GP5 and each item of interest as within-person relationships [33]. The person mean center is calculated for each individual by subtracting their mean response from each of their responses. In the current data, zero indicates average function or symptom severity, positive values indicate worse function or more severe symptoms, and negative values indicate better function or less severe symptoms.

#### Cumulative linked mixed model

The CLMM is a mixed effects model that allows for the modeling of ordinal data, such as the FACT-GP5. The CLMM approach was used because it: (1) allows us to look at the direction and magnitude of relationships between the FACT-GP5 and variables we expect to have similar trajectories (i.e., ECOG performance status, CTCAE grade, other FACT items), which provides evidence for responsiveness of the FACT-GP5; (2) avoids violating assumptions of homoscedasticity, normality of the residuals, and equal distance between response options; and (3) allows for accounting of within-person variability in individuals’ trajectories [34–36]. Each of these features was important, as the FACT-GP5 was nested, did not demonstrate homoscedasticity and normality of the residuals, and had a strong floor effect. To address Aim 2 and understand the impact of missing data on the results, pattern mixture models, a sensitivity analysis technique used to model different missingness patterns [37–41], were also fit.

#### Model building

Following the guidance of Curran and Hussong [42], Curran et al. [43], and Duncan and Duncan [44], we used the model-building process summarized in the supplementary materials in Table A1. To decide when to include random effects, we used guidance from Burnham and Anderson. If the inclusion of random effects improved model fit via a decrease in the AIC of at least 10, random effects were included [45].

#### Pattern mixture model sensitivity analyses

Sensitivity analyses for the impact of missing data on each final model, as determined by the model-building process, were conducted using a pattern mixture model approach. Pattern mixture models allow for exploration of how different missingness patterns impact the interpretation of the results [40]. Choosing which patterns to model depends on several factors: (1) The research question. (2) If there are adequate observations per pattern. (3) Model convergence and interpretability. For longitudinal data, the number of possible patterns is determined by calculating 2^T^, where T is the number of timepoints [41]. Because we had seven timepoints, we had 128 potential missingness patterns to model. To ensure each missingness pattern had adequate observations and to aid in model convergence and interpretability, we chose to examine seven total “patterns”, the number of missing assessments for a participant (zero to six) [41]. Number of missing assessments was added as a continuous predictor to each of the four models, as well as interactions between number of missing assessments and all other predictors in a given model.

## Results

### Descriptive statistics

Baseline characteristics of participants are reported in Table 1. At baseline, patients had a median age of 63 years (range 34–91). Patients identified as Asian (*n* = 8), Black (*n* = 24), Other (*n* = 35), and White (*n* = 551). Eleven patients identified as Hispanic. Around half of the patients (*n* = 332) had a baseline ECOG performance status of 0, around 40% (*n* = 258) had a baseline ECOG performance status of 1, and a small number of patients (*n* = 28) had a baseline ECOG performance status of 2. Descriptive statistics for FACT items, temporally aligned CTCAE grade, performance status, and FACT compliance at each timepoint can be found in Table 2.

**Table 1:** Baseline patient characteristics

	Overall (*N* = 618)
**Age (years)**	
Mean (SD)	62.9 (11.0)
Median [Min, Max]	63.0 [34.0, 91.0]
**Race**	
White	551 (89.2%)
Black	24 (3.9%)
Asian	8 (1.3%)
Other	35 (5.7%)
**Hispanic**	
Yes	11 (1.8%)
No	607 (98.2%)
**Baseline ECOG performance status**	
0 (normal activity)	332 (53.7%)
1 (restricted activity)	258 (41.7%)
2 (in bed > = 50% of the time)	28 (4.5%)
**Measurable disease**	
Yes	481 (77.8%)
No	137 (22.2%)

*ECOG = Eastern Cooperative Oncology Group, FACTGP-5 = Functional Assessment of Cancer Therapy – General Population Item 5

**Table 2:** Descriptive summary of selected items from FACT, CTCAE grade, and ECOG performance status over time

Variable	Baseline	Month 1	Month 2	Month 3	Month 4	Month 5	Month 6
*Treatment Bother* *(FACT-GP5)*							
* n*	520	554	533	392	332	263	228
n (*%*)							
0 (Not at all)	360 (69.2)	334 (60.3)	321 (60.2)	245 (62.5)	214 (64.4)	180 (68.4)	154 (67.5)
1 (A little bit)	74 (14.2)	122 (22.0)	111 (20.8)	77 (19.6)	65 (19.6)	59 (22.4)	43 (18.9)
2 (Somewhat)	57 (11.0)	55 (9.9)	60 (11.3)	42 (10.7)	38 (11.4)	18 (6.8)	19 (8.3)
3 (Quite a bit)	20 (3.8)	28 (5.1)	30 (5.6)	19 (4.8)	11 (3.3)	6 (2.3)	9 (3.9)
4 (Very much)	9 (1.7)	15 (2.7)	11 (2.1)	9 (2.2)	4 (1.2)	0 (0.0)	3 (1.3)
Mean (SD)	1.55 (0.95)	1.68 (1.02)	1.68 (1.01)	1.65 (1.01)	1.57 (0.91)	1.43 (0.72)	1.53 (0.90)
Median	1	1	1	1	1	1	1
*Pain* *(FACT-GP4)*							
* n*	585	559	534	391	334	269	228
Mean (SD)	2.40 (1.18)	2.28 (1.18)	2.33 (1.16)	2.26 (1.13)	2.33 (1.19)	2.07 (1.10)	2.12 (1.12)
Median	2	2	2	2	2	2	2
*Hot Flashes* *(FACT-ES1)*							
* n*	584	566	539	394	338	266	230
Mean (SD)	2.16 (1.25)	2.12 (1.20)	2.12 (1.21)	2.14 (1.15)	2.00 (1.11)	1.99 (1.12)	2.01 (1.12)
Median	2	2	2	2	2	2	2
*Nausea* *(FACT-GP2)*							
* n*	586	565	539	396	334	268	228
Mean (SD)	1.38 (0.81)	1.47 (0.92)	1.40 (0.82)	1.43 (0.88)	1.33 (0.74)	1.35 (0.81)	1.32 (0.78)
Median	1	1	1	1	1	1	1
*Lack of Energy* *(FACT-GP1)*							
* n*	591	567	542	397	336	272	230
Mean (SD)	2.52 (1.17)	2.53 (1.17)	2.50 (1.13)	2.36 (1.10)	2.32 (1.07)	2.18 (1.08)	2.23 (1.09)
Median	2	2	2	2	2	2	2
*CTCAE Grade*							
* n*	596	573	544	400	341	276	234
Mean (SD)	0.12 (0.36)	0.41 (0.72)	0.43 (0.68)	0.50 (0.74)	0.54 (0.76)	0.54 (0.75)	0.55 (0.74)
Median	0	0	0	0	0	0	0
*ECOG Performance Status*							
* n*	618	604	578	420	356	278	245
Mean (SD)	1.51 (0.58)	1.56 (0.64)	1.61 (0.73)	1.53 (0.64)	1.55 (0.67)	1.51 (0.66)	1.46 (0.58)
Median	1	1	2	1	1	1	1

* FACT = Functional Assessment of Cancer Therapy, CTCAE = Common Terminology Criteria for Adverse Events, ECOG = Eastern Oncology Cooperative Group. Visits occur at monthly intervals

### Model 1 - trajectory of the FACT-GP5

**Fig. 1:**
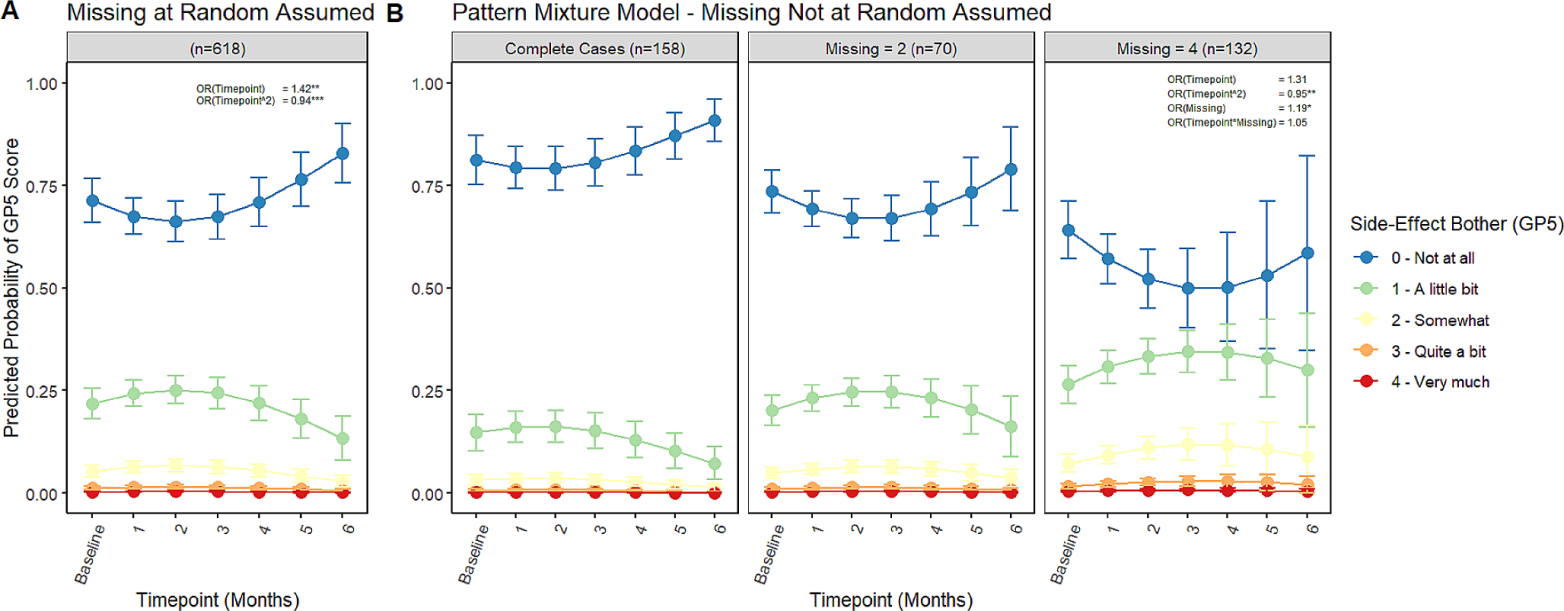
Cumulative linked mixed model (CLMM) predicted probabilities of FACT-GP5 score over time. *p < .05, **p < .01, ***p < .001. FACT = Functional Assessment of Cancer Therapy. Predicted probabilities from CLMM (cumulative linked mixed model) under missing at random assumption and under pattern mixture model. Probability of greater side-effect bother via the FACT-GP5 demonstrated significant linear and quadratic trajectories over time. Under the pattern mixture model sensitivity analyses, the linear trend was no longer significant, and the number of missing assessments demonstrated a positive, significant relationship with level of treatment bother

The best-fitting model for the trajectory of the FACT-GP5 is reported in Table 3. The FACT-GP5 demonstrated a significant linear and quadratic trajectory over the six-month period. Patients had increasing odds of greater side-effect bother via the FACT-GP5 (*OR = 1.42*,* 95% CI = 1.12*,* 1.79)* as time progressed. The predicted probability of greater levels of side-effect bother via the FACT-GP5 peaked at month 2 and then decreased, as shown in Fig. 1A. Results for other FACT and clinician-reported items, using the same model building process, can be found in the supplementary materials in Tables A2 and A3 and in Figures A1 and A2.

**Table 3:** Trajectory of FACT-GP5 side-effect bother

	FACT-GP5 (MAR Assumed)		FACT-GP5 (Pattern Mixture Model, MNAR Assumed)	
	OR (95% CI)	*p*	OR (95% CI)	*p*
*Coefficients*				
Timepoint	1.42 (1.12, 1.79)	0.003**	1.31 (0.99, 1.75)	0.06
Timepoint^2	0.94 (0.92, 0.97)	< 0.001***	0.95 (0.92, 0.98)	0.003**
Missing	NR	NR	1.19 (1.03, 1.38)	0.02*
Missing*Timepoint	NR	NR	1.05 (0.99, 1.10)	0.09

*p < .05, **p < .01, ***p < .001. FACT = Functional Assessment of Cancer Therapy, MAR = Missing at Random, MNAR = Missing Not at Random, NR = Not Relevant. When the FACT-GP5 is assumed to be missing at random, the best-fitting CLMM (cumulative linked mixture model) demonstrates a significant linear and quadratic trajectory of the probability of greater side-effect bother over time. However, after sensitivity analyses using a pattern mixture model approach, (1) there is a positive relationship between number of missing assessments and level of side-effect bother and (2) the linear term is no longer significant, both indicating a violation of the MAR assumption

Including a quadratic random effect in the model resulted in non-convergence. The results suggest there is no between-person variance in how patients’ level of side-effect bother changes quadratically over time, but there is between-person variance in the general linear trajectory of side-effect bother. Note that we were able to successfully model the trajectory without accounting for between-person variance through random effects.

### Models 2–3 - FACT-GP5 and clinician-reported measures

**Fig. 2:**
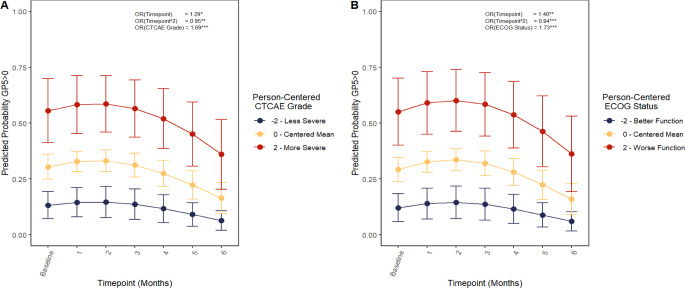
Cumulative linked mixed model (CLMM) predicted probabilities of any treatment bother over time given CTCAE grade or ECOG Status. *p < .05, **p < .01, ***p < .001, FACT = Functional Assessment of Cancer Therapy. CTCAE = Common Terminology Criteria for Adverse Events, ECOG = Eastern Cooperative Oncology Group. Predicted probabilities from CLMM (cumulative linked mixed model). CTCAE Grade is plotted at the centered mean (0), -2, and 2 to demonstrate how a within-person change of 2 levels from their average relates to the predicted probability that GP5 > 0 for a given person. The FACT-GP5 demonstrated significant, within-person, positive relationships with both CTCAE grade and ECOG performance status, controlling for change over time

The best-fitting models for the relationship between the FACT-GP5 and clinician-reported CTCAE grade (Model 2), as well as the relationship between the FACT-GP5 and clinician-reported ECOG performance status (Model 3), are reported in Table 4. For both CTCAE grade and ECOG performance status, there were significant relationships with the FACT-GP5, controlling for time. Within patient, for every one-point increase in CTCAE grade, a patient was 1.69 times more likely (*95% CI = 1.32*,* 2.16*) to select a response option indicating higher side-effect bother on the FACT-GP5. For every one-point increase in ECOG performance status, a patient was 1.73 times more likely to select a response option indicating higher side-effect bother on the FACT-GP5 (*95% CI = 1.32*,* 2.26*). In Fig. 2, these relationships are plotted, examining the predicted probabilities that a patient reports some level of side-effect bother (FACT-GP5 > 0) over time, given deviations from their mean-centered CTCAE grade or mean-centered ECOG performance status.

**Table 4:** Within-person relationship between patient-reported treatment tolerability and clinician-reported outcomes, controlling for change over time

	FACT-GP5 <- CTCAE Grade		FACT-GP5 <- ECOG Status	
	OR (95% CI)	*p*	OR (95% CI)	*p*
*Coefficients*				
Timepoint	1.29 (1.02,1.64)	0.04*	1.40 (1.10, 1.77)	0.006**
Timepoint^2	0.95 (0.92, 0.98)	0.002**	0.94 (0.91, 0.97)	< 0.001***
CRO	1.69 (1.32, 2.16)	< 0.001***	1.73 (1.32, 2.26)	< 0.001***

*p < .05, **p < .01, ***p < .001, FACT = Functional Assessment of Cancer Therapy. CTCAE = Common Terminology Criteria for Adverse Events, ECOG = Eastern Cooperative Oncology Group, CRO = Clinician-reported outcome. CROs were person mean-centered. Results from a CLMM (Cumulative Linked Mixed Model). Controlling for change over time, the FACT-GP5 demonstrated significant, positive, within-person relationships with CTCAE grade and ECOG performance status

### Model 4 - FACT-GP5 and FACT side effect items

**Fig. 3:**
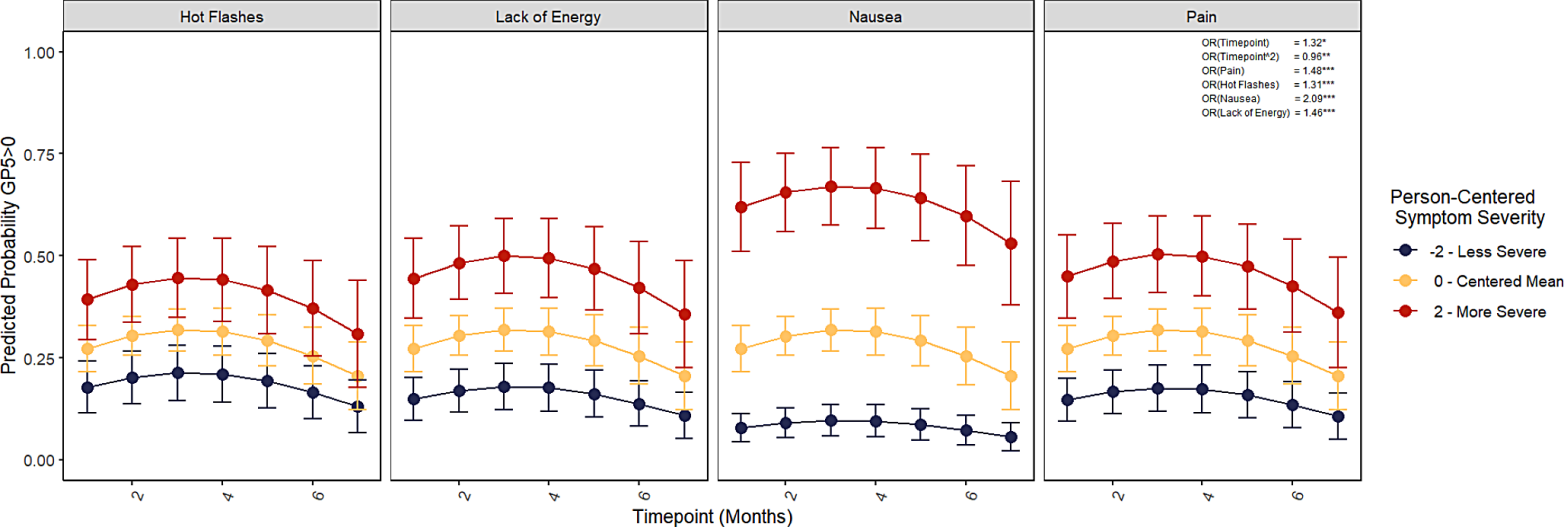
Cumulative linked mixed model (CLMM) predicted probabilities of any treatment bother over time for each FACT item. *p < .05, **p < .01, ***p < .001, FACT = Functional Assessment of Cancer Therapy. Predicted probabilities from CLMM (cumulative linked mixed model). Each FACT symptom is plotted holding all other symptoms in the model constant at the person-centered mean. Symptom severity is plotted at the centered mean, -2, and 2 to demonstrate how a within-person change of 2 levels relates to the predicted probability that GP5 > 0 for a given person. The FACT-GP5 demonstrated significant, within-person, positive relationships with each of the FACT items, controlling for change over time and each of the other side-effect items

The best-fitting model for the relationship between the FACT-GP5 and FACT side-effect items are presented in Table 5. Controlling for time and each other side effect included in the model, if a patient reported higher than their average level of pain (FACT-GP4), nausea (FACT-GP2), or lack of energy (FACT-GP1), they were more likely (*OR = 1.31–2.09*) to report higher levels of side-effect bother via the FACT-GP5, with nausea having the strongest association with bother (*OR = 2.09*,* 95% CI = 1.74*,* 2.51).* Figure 3 shows the predicted probabilities that a patient reports some level of side-effect bother (FACT-GP5 > 0) over time given severity of each side-effect.

**Table 5:** Relationship between treatment bother and FACT symptom items

	FACT-GP5 <- FACT Symptom Items (MAR Assumed)		FACT-GP5 <- FACT Symptom Items (Pattern Mixture Model, MNAR Assumed)	
	OR (95% CI)	*p*	OR (95% CI)	*p*
*Coefficients*				
Timepoint	1.32 (1.04, 1.69)	0.02*	1.36 (1.00, 1.83)	0.05*
Timepoint^2	0.96 (0.93, 0.99)	0.009**	0.96 (0.92, 0.99)	0.01**
Pain	1.48 (1.27, 1.71)	< 0.001***	1.55 (1.26, 1.90)	< 0.001***
Hot Flashes	1.31 (1.12, 1.54)	< 0.001***	1.44 (1.17, 1.79)	< 0.001***
Nausea	2.09 (1.74, 2.51)	< 0.001***	1.61 (1.23, 2.11)	< 0.001***
Lack of Energy	1.46 (1.26, 1.69)	< 0.001***	1.42 (1.16, 1.75)	< 0.001***
Missing	NR	NR	1.34 (1.15, 1.58)	< 0.001***
Missing*Visit	NR	NR	1.00 (0.95, 1.06)	0.80
Pain*Missing	NR	NR	0.98 (0.89, 1.07)	0.60
Hot Flashes*Missing	NR	NR	0.95 (0.86, 1.05)	0.30
Nausea*Missing	NR	NR	1.15 (1.03, 1.28)	0.01*
Lack of Energy*Missing	NR	NR	1.02 (0.94, 1.11)	0.65

*p* < .01, **p < .01, ***p < .001, MAR = Missing at Random, MNAR = Missing Not at Random, FACT = Functional Assessment of Cancer Therapy, NR = Not relevant, FACT predictors were person mean-centered. Results from a CLMM (Cumulative Linked Mixed Model). Assuming the FACT-GP5 is MAR and controlling for change over time and other each other symptom, the FACT-GP5 demonstrated significant, positive, within-person relationships with FACT symptom items. Sensitivity analyses demonstrate that except for nausea, these relationships are not impacted by the number of missing assessments

### Pattern mixture model sensitivity analyses

Pattern mixture model (PMM) results for Model 1 are presented in Table 3. In Model 1, including the number of missing assessments as a predictor resulted in the linear trajectory of the FACT-GP5 no longer being significant (*OR* = 1.31, *95% CI = 0.99*,* 1.75*). The quadratic trend remained significant (*OR* = 0.95, *95% CI = 0.92*,* 0.98)*. These results indicate that when we control for the number of missing assessments, the overall, linear increase in side-effect bother is not present, but a quadratic trend may still be relevant. There was a positive, significant relationship between number of missing assessments and severity of side-effect bother, indicating that higher numbers of missing assessments were associated with more severe side-effect bother (*OR* = 1.19, *95% CI = 1.03*,* 1.38*). Figure 1B illustrates the levels and trajectories of the FACT-GP5 for different numbers of missing assessments. As patients have greater numbers of missing assessments, they are more likely to report higher side-effect bother.

**Fig. 4:**
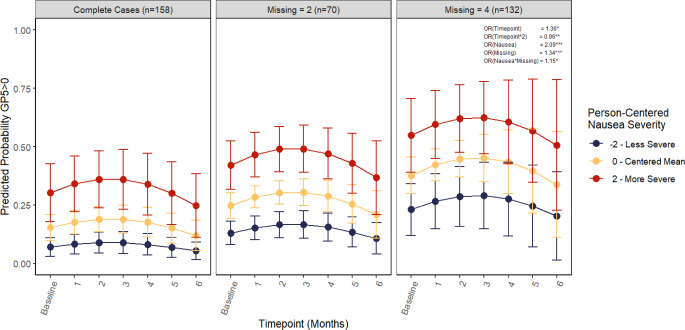
Cumulative linked mixed model (CLMM) predicted probabilities of any treatment bother given nausea severity, by number of missing assessments and controlling for all other side effect items. *p < .05, **p < .01, ***p < .001. Predicted probabilities from pattern mixture sensitivity analyses on CLMM (cumulative linked mixed model). Nausea is plotted holding all other FACT symptoms in the model constant at the person-centered mean. Nausea severity is plotted at the centered mean, -2, and 2 to demonstrate how a within-person change of 2 levels relates to the predicted probability that GP5 > 0 for a given person. There was a significant interaction effect between nausea severity and number of missing assessments, where patients are more likely to report greater treatment bother when they report greater nausea severity and have a greater number of missing assessments

Except for nausea, sensitivity analyses on the relationship between the FACT-GP5 and CTCAE grade (Model 2), ECOG performance status (Model 3), and other FACT side-effect items (Model 4) indicate no impact of missingness on the results. For nausea, there was a significant interaction effect between severity of nausea and number of missing assessments (*OR = 1.15*,* 95% CI = 1.03*,* 1.28).* Trajectories for the relationship between nausea severity and number of missing assessments can be found in Fig. 4 and results are reported in Table 5. Greater numbers of missing assessments are associated with stronger relationships between nausea severity and level of side-effect bother.

## Discussion

From our analyses, we found that patients, *initially*, were more likely to report higher levels of side-effect bother, but *eventually*, as time progressed, an improvement was observed. Because PROMs can be administered at home, monitoring patients via the FACT-GP5 in combination with other measures of side-effect severity may aide in earlier detection of treatment bother, thus allowing for earlier side-effect management.

Additionally, the results suggest positive within-person relationships between level of side-effect bother and severity of other FACT items, as well as ECOG performance status and CTCAE grade, controlling for time, suggesting that the FACT-GP5 is a responsive measure. However, it is difficult to establish a temporal relationship, and thus, we urge caution in making any causal inference based on the observed data from this study.

Finally, based on sensitivity analyses, trajectories of the FACT-GP5 and ECOG performance status, as well as other FACT items appear to be impacted by the patients’ number of missing assessments, indicating that accounting for missing data patterns is important when describing change over time in these measures. However, the relationship between the FACT-GP5 and these symptoms of interest, excluding nausea, were not impacted by the number of missing assessments. This indicates that the responsiveness of the FACT-GP5 to pain, hot flashes, and lack of energy was not impacted by the number of missing assessments in our study, but the responsiveness of the FACT-GP5 to nausea was impacted by the number of missing assessments. As previously stated, modeling of missing data patterns will be important when assessing change in the FACT-GP5. This may be particularly important if nausea is a commonly reported side-effect.

### Limitations

There are some limitations to the current research. The timing and frequency of assessment may not have been sufficient to capture side-effect bother. As typical in clinical trials, patients completed the FACT with a 7-day recall period on the day that they received injections (administered monthly). If side-effects to the injections are more prevalent or severe in the days immediately following administration, this would not be captured by the FACT. It is also possible that a more frequent schedule of assessment would impact missing data analyses.

Next, the way in which CTCAE grade was captured and adjusted to align with the PRO schedule may not have best represented when AEs were occurring. AEs that were not marked as resolved were treated as ongoing, but it is possible there is human-measurement error in recording resolution of an AE.

Additionally, including a random quadratic effect led to convergence issues when modeling the trajectory of the FACT-GP5. Although we cannot know for sure, this was potentially due to strong relationships between linear and quadratic trajectories (i.e., collinearity) and/or lack of between-person variability in the trajectories. That is, patients may show similar changes in the FACT-GP5, quadratically, over time. Fixed effects were reported. Data with greater between-person variability, larger sample sizes, and more timepoints may yield different results.

Finally, although this study aimed to further understand the relationship between commonly occurring treatment-related AEs and overall side-effect bother, it was limited by the PRO data collected in the trial. For example, injection-site pain was identified as a common AE based on CTCAE data, but only general pain, as measured by the FACT-GP4, was measured via PRO in the trial. This highlights the importance of targeted and fit-for-purpose PRO measurement selection.

### Future directions

Assessing the responsiveness of the FACT-GP5 and the impact of missing data in other cancer populations and with differing schedules of assessment will aid in determining the robustness of the FACT-GP5 as a measure of global tolerability. Additionally, it would be useful to compare the FACT-GP5 to PRO-CTCAE measures and not just clinician-reported CTCAE grades to better understand which and what severity of AEs, as perceived by the patient, result in reporting side-effect bother on the FACT-GP5. Finally, a more frequent measurement schedule (e.g., weekly or bi-weekly) would allow for a more granular understanding of the relationship between the FACT-GP5, CTCAE grade, and specific side effects through time series modeling approaches.

Future research should also be focused on studying the optimal way to assess overall treatment tolerability. This study (and others) start with assuming that the FACT-GP5 as a single item may capture this burden. Future studies should both use concept elicitation type qualitative studies to hear how patients describe treatment tolerability and explore through cognitive interview-based studies if one or more items can more fully capture treatment tolerability. If the FACT- GP5 or another set of items are deemed content valid, then placement of the item (set of items) within the PRO assessment should be considered to capture comprehensively the patient experiences. For example, the FACT-GP5 is the fifth item administered with other specific AE questions asked later. It would likely be better to move the FACT-GP5 item to the end to allow the patient to go through all the potential AE experiences.

## Conclusions

The FACT-GP5 as a global item is responsive, allowing for an overall view of the totality of side effect bother in combination with other questionnaires assessing treatment-related symptoms. Sensitivity analyses demonstrate that the severity of side-effect bother is likely related to the amount of missing data a given patient has; however, the responsiveness of the FACT-GP5 is generally robust to missingness. Research on the responsiveness of the FACT-GP5 should be expanded to a diverse range of cancer populations and demonstrate comparisons with a wide range of side-effect items.

## Electronic supplementary material

Below is the link to the electronic supplementary material.

Supplementary Material 1
