# Supplementary material for: The FACT-GP5 as a global tolerability measure: responsiveness and robustness to missing assessments
Source: Qual Life Res. 2024 Jul 24;33(10):2869–80. doi: 10.1007/s11136-024-03740-x (PMC11452438; doi:10.1007/s11136-024-03740-x)
Supplement: Supplementary file 1 — Supplementary Material 1 [file 11136_2024_3740_MOESM1_ESM.docx]

**The FACT-GP5 as a global tolerability measure: responsiveness and robustness to missing assessments**

**Quality of Life Research**

Cara Arizmendi, PhD^a,b^ (0000-0001-5608-7385); Yanyan Zhu, PhD^a^; Maryam Khan, MS^a^; Jonathon Gable, MPA^a^; Bryce B. Reeve, PhD^b^ (0000-0002-6709-8714); Bellinda King-Kallimanis, PhD^c^; Jill Bell, PhD^a^

^a^ AstraZeneca, Oncology Digital Health R&D, Gaithersburg, MD USA

^b^ Center for Health Measurement, Department of Population Health Sciences, Duke University School of Medicine, Durham, NC, USA

^c^ Lungevity Foundation, Chicago, IL USA

**Corresponding author:** Cara Arizmendi, PhD; cara.arizmendi@astrazeneca.com

**Supplementary Materials**

*Table A1. Model-building process*

| ***Model 1 –***  ***FACT-GP5 <- Timepoint*** | ***Term Added*** | | ***Retain term added?*** | |
| --- | --- | --- | --- | --- |
| *Step 1* | *Random Intercept* | | Yes | |
| *Step 2* | *Timepoint* (fixed) | | Yes | |
| *Step 3* | *Timepoint^2^* (fixed) | | If significant at p<.05 | |
| *Step 4* | *Timepoint* (random)  *Timepoint^2^* (random; added if fixed timepoint^2^ term is significant) | | If decrease in AIC ≥ 10 | |
| ***Models 2-4 –***  ***FACT-GP5 <- Predictor*** | ***Starting with Model 1 as base model, term added*** | ***Retain term added?*** | |  |
| *Step 1* | *Predictor* (fixed) | | Yes | |
| *Step 2* | *Predictor* (random) | | If decrease in AIC ≥ 10 | |
| *Step 3* | *Timepoint*Predictor* (fixed) | | If significant at p<.05 | |
| *Step 4* | *Timepoint^2^*Predictor* (fixed; added if Timepoint*Predictor is significant) | | If significant at p<.05 | |
| *Step 6* | *Timepoint*Predictor* (random; added if Timepoint*Predictor is significant) | | If decrease in AIC ≥ 10 | |
| *Step 7* | *Timepoint^2^*Predictor* (random; added if Timepoint^2^*Predictor is significant) | | If decrease in AIC ≥ 10 | |

* *FACT = Functional Assessment of Cancer Therapy, AIC = Akaike Information Criteria.*

| *Table A2. Trajectories of FACT items.* | | | | | | | | |
| --- | --- | --- | --- | --- | --- | --- | --- | --- |
|  |  | | | |  | | | |
|  | **Pain** | | | | **Hot Flashes** | | | |
|  | Estimate | OR (95% CI) | *SE* | *p* | Estimate | OR (95% CI) | *SE* | *p* |
| *Coefficients* |  |  |  |  |  |  |  |  |
| Timepoint | -0.03 | 0.97 (0.91, 1.02) | 0.03 | 0.21 | -0.14 | 0.87 (0.81, 0.93) | 0.04 | <.001*** |
| Timepoint^2 | NR | NR | NR | NR | NR | NR | NR | NR |
|  |  |  |  |  |  |  |  |  |
|  | σ^2^ | *r* |  |  | σ^2^ | *r* |  |  |
| *Random Effects* |  |  |  |  |  |  |  |  |
| Intercept | 6.6 | ---- |  |  | 14.08 | ---- |  |  |
| Timepoint | 0.08 | -0.01 |  |  | 0.15 | -0.39 |  |  |
|  |  |  |  |  |  |  |  |  |
| *Model Fit* |  |  |  |  |  |  |  |  |
| AIC | 7019.92 |  |  |  | 6278.73 |  |  |  |
| ****p<.001. FACT = Functional Assessment of Cancer Therapy. NR = Not Relevant, AIC = Akaike Information Criterion. Results from CLMMs (Cumulative Linked Mixed Models). Except for hot flashes, the symptom-specific FACT items did not show significant change in probability of greater symptom severity over time. Hot flashes demonstrated a negative relationship over time, whereas severity of hot flashes decreased over time.* | | | | | | | | |

| *Table A2. Trajectories of FACT items (cont.)* | | | | | | | | |
| --- | --- | --- | --- | --- | --- | --- | --- | --- |
|  |  | | | |  | | | |
|  | **Nausea** | | | | **Lack of Energy** | | | |
|  | Estimate | OR (95% CI) | *SE* | *p* | Estimate | OR (95% CI) | *SE* | *p* |
| *Coefficients* |  |  |  |  |  |  |  |  |
| Timepoint | 0.01 | 1.01 (0.95, 1.07) | 0.03 | 0.81 | -0.01 | 0.99 (0.83, 1.18) | 0.09 | 0.91 |
| Timepoint^2 | NR | NR | NR | NR | -0.01 | 0.99 (0.97, 1.01) | 0.01 | 0.45 |
|  |  |  |  |  |  |  |  |  |
|  | σ^2^ | *r* |  |  | σ^2^ | *r* |  |  |
| *Random Effects* |  |  |  |  |  |  |  |  |
| Intercept | 6.51 | ---- |  |  | 6.36 | ---- |  |  |
| Timepoint | ---- | ---- |  |  | 0.12 | -0.35 |  |  |
|  |  |  |  |  |  |  |  |  |
| *Model Fit* |  |  |  |  |  |  |  |  |
| AIC | 3986.35 |  |  |  | 7450.38 |  |  |  |
| ****p<.001. FACT = Functional Assessment of Cancer Therapy. NR = Not Relevant, AIC = Akaike Information Criterion. Results from CLMMs (Cumulative Linked Mixed Models). Except for hot flashes, the symptom-specific FACT items did not show significant change in probability of greater symptom severity over time. Hot flashes demonstrated a negative relationship over time, whereas severity of hot flashes decreased over time.* | | | | | | | | |

| *Table A3. Clinician-Reported Outcome Growth Curve Models* | | | | | | | | |
| --- | --- | --- | --- | --- | --- | --- | --- | --- |
|  |  | | | |  | | | |
|  | **CTCAE Grade** | | | | **ECOG Performance Status** | | | |
|  | Estimate | OR (95% CI) | *SE* | *p* | Estimate | OR (95% CI) | *SE* | *p* |
| *Coefficients* |  |  |  |  |  |  |  |  |
| Timepoint | 1.83 | 6.21 (4.65, 8.30) | 0.15 | <.001*** | 0.47 | 1.60 (1.26, 2.03) | 0.12 | <.001*** |
| Timepoint^2 | -0.18 | 0.84 (0.81, 0.86) | 0.02 | <.001*** | -0.05 | 0.95 (0.93, 0.98) | 0.01 | 0.003** |
|  |  |  |  |  |  |  |  |  |
|  | σ^2^ | *r* |  |  | σ^2^ | *r* |  |  |
| *Random Effects* |  |  |  |  |  |  |  |  |
| Intercept | 14.92 | ---- |  |  | 12.45 | ---- |  |  |
| Timepoint | ---- | ---- |  |  | ---- | ---- |  |  |
|  |  |  |  |  |  |  |  |  |
| *Model Fit* |  |  |  |  |  |  |  |  |
| AIC | 3558.82 |  |  |  | 3976.25 |  |  |  |
| ****p<.001. CTCAE = Common Terminology Criteria for Adverse Events, ECOG = Eastern Cooperative Oncology Group, AIC = Akaike Information Criterion. Results from CLMMs (Cumulative Linked Mixed Models).*  *Both CTCAE grade and ECOG performance status show significant linear and quadratic trajectories of the probability of greater severity over time.* | | | | | | | | |

**Figure A1** Predicted probabilities of FACT item scores over time


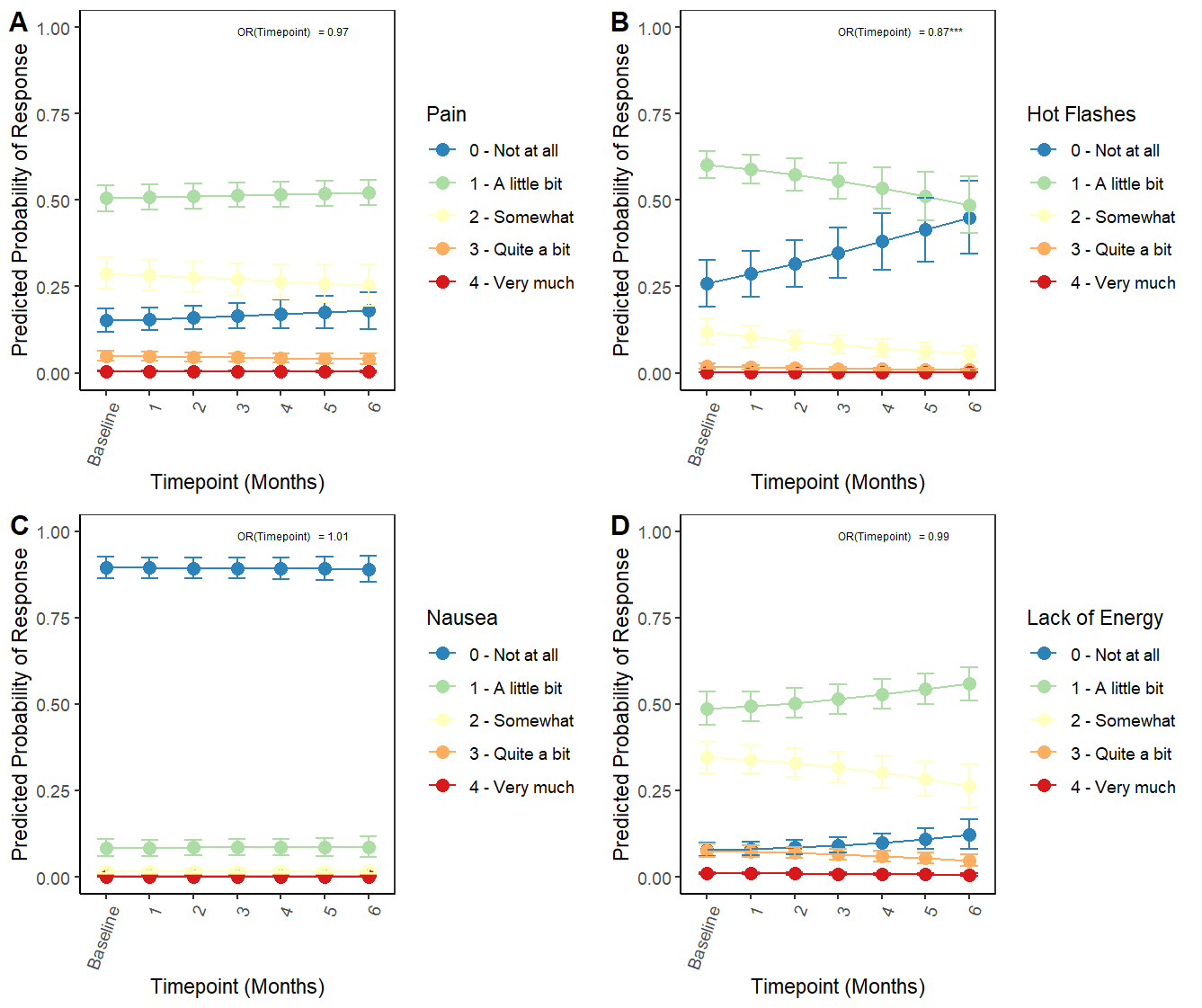


****p<.001, FACT = Functional Assessment of Cancer Therapy. Predicted probabilities from a CLMM (Cumulative Linked Mixed Model).* *Except for hot flashes, these FACT items did not demonstrate significant change over time.*

**Figure A2** Predicted probabilities of clinician-reported item scores over time


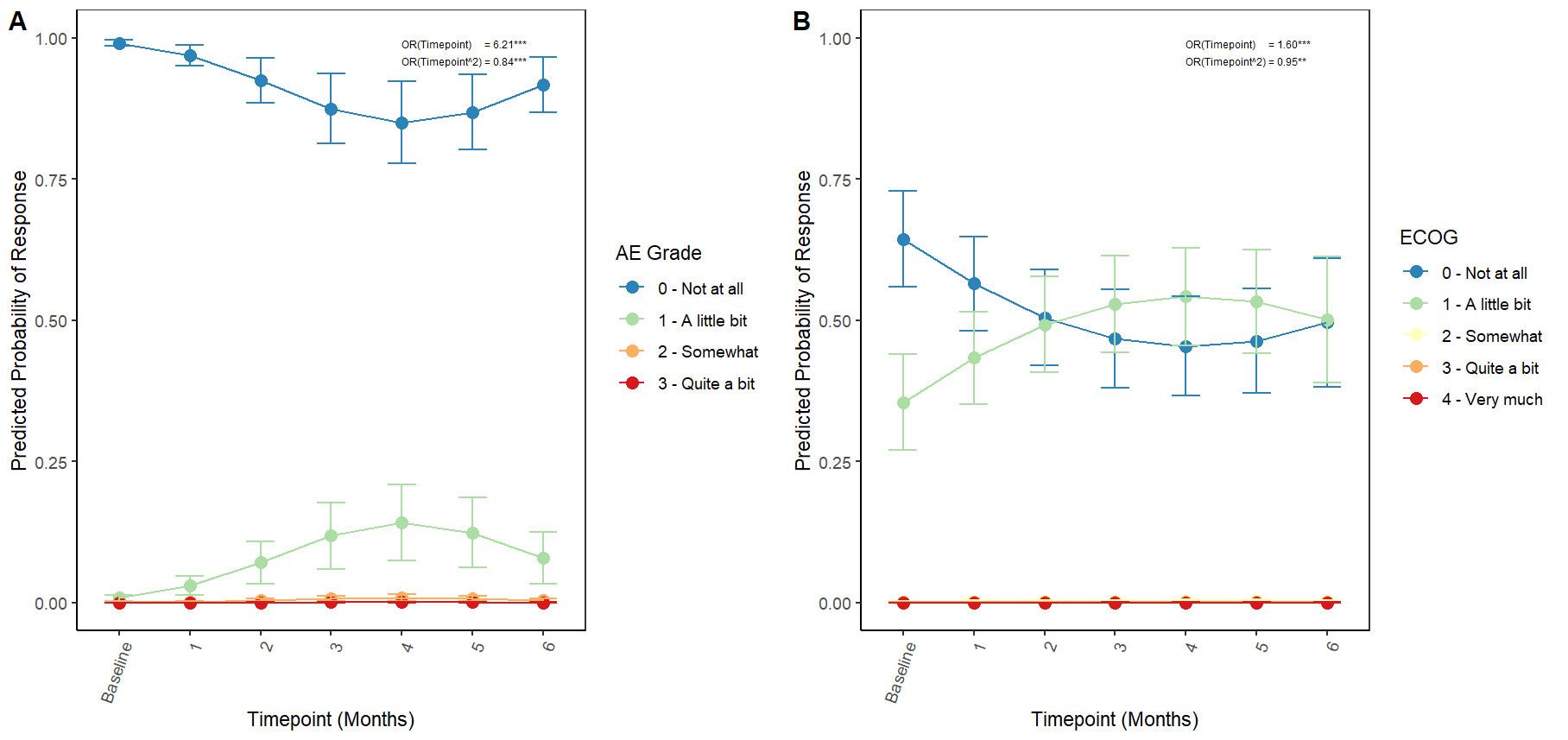


****p<.001. CTCAE = Common Terminology Criteria for Adverse Events, ECOG = Eastern Cooperative Oncology Group.*  *Predicted probabilities from a CLMM (Cumulative Linked Mixed Model).* *Both CTCAE grade and ECOG performance status show significant linear and quadratic trajectories over time.*
